# The Association between Indoor Air Quality and Adult Blood Pressure Levels in a High-Income Setting

**DOI:** 10.3390/ijerph15092026

**Published:** 2018-09-17

**Authors:** Krassi Rumchev, Mario Soares, Yun Zhao, Christopher Reid, Rachel Huxley

**Affiliations:** 1 School of Public Health, Curtin University, Perth, WA 6148, Australia; m.soares@curtin.edu.au (M.S.); Y.Zhao@curtin.edu.au (Y.Z.); Christopher.Reid@curtin.edu.au (C.R.); R.Huxley@latrobe.edu.au (R.H.); 2 School of Public Health and Preventive Medicine, Monash University, Melbourne, VIC 3800, Australia; 3 College of Science, La Trobe University, Melbourne, VIC 3086, Australia

**Keywords:** indoor air quality, particulate air pollution, blood pressure, heart rate, Australia

## Abstract

**Background:** Indoor air pollution is still considered one of the leading causes of morbidity and mortality worldwide. We aimed to investigate the potential association between indoor particulate matter (PM) and fasting clinic blood pressure in adult Australians. **Methods:** Sixty-three participants residing within the Perth metropolitan area were studied. Participants were aged between 18 and 65 years and free of major medical conditions. We conducted 24-h monitoring of residential PM concentrations, including the size fractions PM1, PM2.5, PM4, and PM10. All participants attended a clinical assessment at Curtin University following a 10–12 h overnight fast. **Results:** In this study we found that PM1 and PM2.5 were significantly associated with heart rate: a one interquartile range (IQR) increase in PM1 or PM2.5 was associated with a 4–6 beats per minute (bpm) increase in heart rate. Both PM10 and total PM exposure had a significant impact on systolic blood pressure (SBP): a one IQR increase in PM10 and total PM were associated with a 10 mmHg (95% CI: 0.77–20.05) and 12 mmHg (2.28–22.43 mmHg) increase in SBP, respectively. **Conclusion:** The study findings provide additional support to the thesis that indoor air pollution is an important modifiable factor in the risk of hypertension.

## 1. Introduction

Despite decades of progress in both the prevention and the management of individuals with elevated blood pressure (BP), suboptimal BP remains the single, most important contributor to the global burden of cardiovascular diseases [1,2]. In recent years, there has been a growing appreciation of the role of poor air quality in health outcomes, especially in relation to respiratory and cardiovascular diseases (CVD). In 2014, the World Health Organization identified air pollution as the largest single environmental health risk factor, responsible for an estimated 3.7 million deaths in 2012 [3].

Particulate air pollution is a complex mixture of all the solid and liquid particles suspended in air and can include both organic and inorganic particles, such as dust, pollen, soot, and smoke. The particulate matter (PM) in air is typically classified by size into coarse (PM_10_; <10 μm mean aerodynamic diameter), fine (PM_2.5_; <2.5 μm diameter), and ultrafine (UFP; <0.1 μm diameter) fractions. The main source of indoor particles is from outdoor air, however indoor activities such as cooking or indoor combustion [4]; smoking [5]; vaping [6]; secondary formation processes [7] and dust resuspension are also considered as significant contributors to indoor aerosols. Ambient aerosols in urban environments usually originate predominantly from fossil fuel burning, car emissions, resuspension, and chemical and thermodynamic processes, but can also come from long range transport [8]. Due to their diverse sources, airborne particles span a large size range, from a few nanometers to tens of micrometers in diameter. However, most studies to date have identified PM_2.5_ as having the strongest adverse effects on health, particularly vascular outcomes [9,10,11]. The mechanisms underlying the relationship between exposure to particulate matter and markers of vascular health remain unclear but increased oxidative stress and inflammation have been reported as possible pathways mediating the relationship [10,12,13]. In addition, recent research has demonstrated that PM also affects the cardiovascular system directly by entry into the systemic circulation [14]. This process causes myocardial dysfunction through reactive oxygen species production, calcium ion interference, and vascular dysfunction. 

Traditionally, epidemiological studies have focused on the associations between outdoor air pollution and cardiovascular (CV) health outcomes. However, there is increasing evidence that poor indoor air quality (IAQ) may also be associated with adverse vascular and respiratory outcomes. It has been estimated that in high-income countries, individuals spend up to 90% of their time indoors, of which nearly two-thirds is spent in their primary residence; thus, indoor air quality may be a potential risk factor for human health [15,16]. According to Morawska et al. [17], between 10–30% of the global burden of disease from PM exposure is attributable to indoor-generated particles. However, most of the studies to date have been conducted in lower- and middle-income countries where levels of air quality—both indoors and outdoors—are significantly poorer that in high-income countries. According to the World Bank, high-income economies are those with a per capita gross national income (GNI) of $12,056 or more, which includes Australia [18]. To date, few studies have examined the relationship between indoor-generated particulate matter and markers of vascular health in a high-income setting. The objective of this study was to investigate the potential association between different indoor PM size fractions and both BP and heart rate (HR) outcomes in adult Australians.

## 2. Methods 

The study included volunteers (*n* = 63) residing within the Perth metropolitan area, aged between 18 and 65 years, who were free of major medical conditions including type 2 diabetes, cardiovascular disease and cancer. We applied the convenience sampling method, with study subjects recruited via advertising through radio and pamphlets distributed at the Curtin University campus. The Human Research Ethics Committee at Curtin University provided approval for the study.

### 2.1. Measurement of Indoor Air Quality

In this study we examined the potential association between chronic exposures, not acute exposures, to air pollutants and a chronic condition of altered resting BP. In a similar air quality study, 24-h measurement was considered sufficient time to capture long term domestic exposure to air pollutants [19,20]. This sampling duration was applied in the current study. Real-time measurements of PM concentrations were conducted using a TSI DustTrak™ Aerosol Monitor Model 8530 (TSI Inc., Shoreview, MN, USA)), with a size-selective inlet conditioner. The monitor operated at a flow rate of 1.7 L/min. Monitoring was conducted over a 24-h period in participants’ living room at breathing height and concentrations of particles with different sizes including PM_10_, PM_4_, PM_2.5_ and PM_1_ were recorded. 

In addition to the regular annual factory calibration, the DustTrak™ was custom calibrated using the integral 37 mm filter at some selected site locations, to determine the gravimetric concentration. The custom calibration factor was reused at all measurement sites. A TinyTag Ultra 2TM data logger (Gemini Data Loggers (UK) Ltd., Chichester, West Sussex, UK) was used to measure indoor temperature and relative humidity. All measurements were conducted during weekdays and participants were asked to maintain their usual behavioral patterns. 

### 2.2. Measurement of Covariates

A modified structured questionnaire, based on the questionnaire of the American Thoracic Society for respiratory symptoms [21], and applied in other studies [22,23,24], was used to elicit the required data. The questionnaire comprised two parts: (a) individual data on the participant’s age, education, gender, and some general questions about health, physical activities and diet, and (b) data on the participant’s home environment, including exposure to cigarette smoke and type of heating and cooling present in the home. 

### 2.3. Assessment of Heart Rate and Blood Pressure

All participants were invited to come to Curtin University for clinical assessment. Following a 10–12 h overnight fast, minimum 8 h sleep and abstinence from alcohol and from nicotine (for those who smoked), participants arrived at the clinical suite in the School of Public Health where body weight and composition was determined using a bio-impedance analysis (BIA) instrument (Inbody 3.0, InBodyCo. Ltd., Seoul, Korea). Following a mandatory 30 min rest in bed, with an appropriate sized BP cuff in place on exposed arm, BP and HR (Omron, Kyoto, Japan), were measured in triplicate and the lowest two readings were averaged. All measurements followed a standard protocol, used in previous studies [25,26]. The order of measurements was random, with participants either having their clinical parameters measured first, followed by the indoor air pollution of their homes, or vice versa. The interval between these measures was at most 3 weeks. 

### 2.4. Statistical Analysis

In this study, the main outcome variables were HR and systolic (SBP) and diastolic (DBP) blood pressure (mmHg). The exposure variables of interest were indoor measurements of PM_1_, PM_2.5_, PM_4_, and PM_10_. Descriptive statistics were produced to describe the profile and characteristics of participants and to summarize the outcome variables and the exposure/independent variables of interest. All outcome variables were assessed for normality and the natural logarithm transformation was applied to HR. The natural logarithm transformed HR was used in the multiple regression analysis. The estimated coefficients, which correspond to the expected geometric mean of the HR measurements, were then back transformed using the exponential function to calculate relevant changes in HR. Untransformed SBP and DBP were used in all regression modellings. The geometric mean changes in HR and the mean changes in BP corresponding to a one interquartile range (IQR) increase in indoor PM concentrations are reported, along with their 95% confidence interval (CI) (corresponding percentage (%) changes are reported in the supplementary figures). Multivariable linear regression analysis was conducted to assess the association between particulate air pollution and both BP and HR, controlling for age, gender, smoking (in house), body mass index (BMI), high BP status (yes vs. no prior to recruitment), use of prescription medicines (yes vs. no) and McAuley’s Insulin Sensitivity Index (ISI; an index based on insulin and triglyceride levels) for all subjects. Subgroup analyses used the same list of covariates, with high BP status and smoking removed accordingly.

In this study a negative confounding effect on the association between PM concentration and the outcome variables (in particular HR) was observed. To identify the variable/s with the potential to cause this effect, multivariable linear regression analysis was conducted multiple times with a different variable or combination of variables removed in each model. The above modelling procedures and the assessments of negative confounding were repeated separately for a subgroup comprised of the non-hypertensive subjects (*n* = 41). All statistical analyses were performed using SPSS version 22 (IBM, Armonk, NY, USA).

## 3. Results 

A total of 63 subjects (55% women) with a mean age of 61 years (SD = 9) were recruited into the study. Almost half of the participants (49%, *n* = 31) reported taking blood-pressure-lowering medication. The mean SBP and DBP values were 130.3 mmHg (SD = 17.7 mmHg) and 75.1 mmHg (SD = 10.0 mmHg), respectively. Mean HR was 63.3 beats per minute (bpm; SD = 12.6 bpm). Additional baseline characteristics for study participants are shown in Table 1. The occupants of 38 (60.3%) households were exposed to mean PM_2.5_ concentrations that were within the WHO Guideline (≤25 µg/m^3^), whereas 20 households (32%) had a mean PM_2.5_ that exceeded the guideline value and five houses showed no detectable PM_2.5_. Summary data on the levels of indoor particulate matter, temperature and relative humidity are presented in Table 2. Four participants (6%) reported current smoking of cigarettes in the house. In these households the median PM_10_ and total PM levels were significantly (*p* = 0.002) higher compared with the smoke-free households: 89 µg/m^3^ and 153 µg/m^3^ versus 29 µg/m^3^ and 62 µg/m^3^, respectively. In addition, total PM levels were significantly higher (*p* < 0.05) in households located less than 300 m from a major road than in those situated away from a main thoroughfare: 67 µg/m^3^ versus 48 µg/m^3^, respectively.

### Association between Levels of Indoor Particulate Matter with Heart Rate and Blood Pressure

In the crude-model adjusted only for age and gender, there was a positive trend observed in HR with increasing exposure to each PM size fraction: an increase in one IQR for each of the exposure measures was associated with an approximate increase in HR of 3–4 bpm. In the analysis adjusted for age, gender, smoking, BMI, high BP, use of prescription medicines and McAuley’s ISI, there was a significant association of PM_1_ and PM_2.5_ with HR such that a one IQR increase in exposure was associated with an approximately 4 bpm increase in HR (Table 3).

When the analysis was restricted to those who were not diagnosed as hypertensives nor reported taking blood-pressure-lowering medication (*n* = 41), the relationship was strengthened, as a one IQR increase in PM_1_ and PM_2.5_ was associated with a 6 bpm higher HR (Table 4). Excluding the current smokers from the analysis (*n* = 4) did not significantly affect the results. 

There were weak positive associations between levels of indoor particulate matter and both SBP and DBP in the adjusted model but none were statistically significant (Table 3). When the data was analyzed for only those who were not diagnosed as hypertensives nor taking blood-pressure-lowering medication (*n* = 43), the associations were much stronger. The most pronounced effect was observed for total PM, a one IQR increase in total PM was associated with a 13 mmHg increase in SBP ((95% CI: 4.07–22.81 mmHg) (Table 4)). 

In addition, Supplementary Figures S1 and S2 show the percent change in each of the outcome variables associated with exposure to a one IQR increase in PM concentration, for the adjusted model. The data for the analysis of all participants is shown in Supplementary Figure S1, the data for the analysis of only participants that did not report a diagnosis of high BP is shown in Supplementary Figure S2, based on these figures, each IQR increase exposure in PM_1_ and PM_2.5_ was significantly associated with an approximate 7–8% elevation in HR on average, which was a finding consistent across subgroups. 

## 4. Discussion

The adverse effects of outdoor air pollution on CVD outcomes have been widely studied. In comparison, there is a paucity of information regarding the effect of indoor air quality on CVD health, particularly in high-income settings. In this adult study population, 24-h measures of exposure to indoor PM, and in particular PM_1_ and PM_2.5_, were strongly associated with vascular risk markers, which is in agreement with previous findings [12,27,28,29]. We found that PM_1_ and PM_2.5_ were positively associated with HR: a one IQR increase in PM_1_ or PM_2.5_ was associated with a 4–6 bpm increase in HR. Brook [10] and colleagues have also postulated that particulate air pollutants can trigger elevation in HR. Similar results were reported by Zhao and colleagues [30], who established a significant association between PM and increased HR among the Chinese population. Large-scale epidemiological studies have demonstrated a positive relationship between HR and vascular risk. In the HARVEST study [31], patients whose HR was persistently high during the study period of six years had a doubled risk of developing hypertension. Fox and colleagues [32] showed that a resting HR of >70 bpm was associated with increased cardiovascular mortality as well as an increase in hospitalization due to heart failure or myocardial infarction. 

The effects of PM on BP were somewhat equivocal but supported the existence of a positive relationship between exposure to particular air pollution and higher SBP and DBP. Dianat and colleagues [33] concluded that PM_10_ had harmful effects on the heart and on BP in healthy rats, likely due to increased oxidative stress. In another recent study, Zhang and colleagues [34] demonstrated that a 10 μg/m^3^ increase in PM_2.5_ concentration was associated with a 0.5 mmHg increase in SBP. In the current study, a one IQR increase in PM_10_, was associated with a 10 mmHg higher SBP among non-hypertensive participants who did not take BP medications, which is consistent with the findings of a study conducted in Detroit [35]. Total PM appeared to also be a significant contributor to elevated levels of SBP, as a one IQR increase in total PM was associated with a 13 mmHg (95% CI: 2.28–22.43) higher SBP. These results clearly demonstrate that there is a wide range in the magnitude of BP elevation between subjects and therefore susceptible individuals may respond with larger degrees of BP elevation than the population mean.

Recent research has shown that PM can raise BP, and this effect may even persist chronically when individuals reside in more polluted regions [36,37]. This is consistent with the findings of Brook and colleagues [38] who provided evidence that long term PM exposure can promote the development of hypertension. This suggests that not only can short-term PM exposures potentially cause an acute increase in BP, but chronically inhaling second-hand smoke can increase the risk for chronic hypertension. In a more recent study in China, Lin and colleagues [39] demonstrated that an IQR increase of PM_2.5_ (19.1 μg/m^3^) was associated with the increase of 1.90 mmHg in SBP and 0.68 mmHg in DBP. It should be noted that, not all studies have positive results [40,41]. The discrepancies between previous studies may result from PM exposure mischaracterizations, the protective effects of medications taken by some participants, possible lack of adjustments for other confounders, and inaccurate determinations of BP [42]. In this study we established negative confounding for the PM_1_ and PM_2.5_ outcomes (Table 3 and Table 4). The statistical analysis indicated that McAuley’s ISI, with the highest missing rate (*n* = 15, 23.81%), was the potential variable causing this phenomenon. McAuley’s ISI is a measure of insulin sensitivity, and according to the study outcomes, the index was mainly affected by fine particulate matter concentrations. We propose that high concentrations of PM_1_ and PM_2.5_ may result in low a McAuley’s ISI that, in turn, can lead to increased HR. Confirmation of this proposed pathway can be a focus of future studies. 

An important and novel aspect of the current study, which suggests an adverse association between exposure to indoor particulate matter and vascular risk factors, is that the data were derived from a population living in high-income setting where air quality is usually very good by international standards. Most previous studies of indoor air quality on health outcomes have been conducted in lower and middle-income countries where exposure to high levels of indoor air particulate matter is common due to poor indoor ventilation, heating and cooking practices. In this study, median levels of PM_2.5_ and PM_10_ were within the Australian National Environmental Protection Measures (NEPM) 24-h exposure standards of 25 µg/m^3^ and 50 µg/m^3^, respectively. Study participants, who lived in houses less than 300 m from busy roads or that allowed smoking inside were exposed to PM concentrations that exceeded the NEPM standards. 

In the context of cardiovascular (CV) risk factors, BP is strongly and directly related to CV mortality [43] and is ranked as the leading risk factor for the global diseases burden, having accounted for more than nine million deaths in 2010 [44]. Furthermore, Lim [44] stated that while higher levels of both PM and BP are each individually linked to premature morbidity and mortality, a biological interconnection between these two leading risk factors could represent an unprecedented threat to global public health. 

### Limitations

This pilot study on a small sample was conducted over a relatively short duration. Hence seasonal variations in IAP/OAP (outdoor air pollution) are embedded in these results. Our significant findings suggest a particularly large effect in comparison to data from other studies. In part, this could have arisen from unmeasured indoor exposure data on ozone, ultrafine particles, and nitrogen dioxide. The latter are known to independently influence CVD risk and correlate strongly with PM. Hence their non-measurement would possibly have biased our study outcomes [45]. In addition, a single 24-h measure may not represent the usual indoor air particle concentration, and this is considered a study limitation. Another limitation acknowledged by the authors is that the chemical composition of the specific PM constituents responsible for the observed adverse effects on HR and BP were not identified.

## 5. Conclusions

In this observational study we demonstrate significant positive associations between several measures of indoor PM and both HR and BP, after accounting for number of covariates including age, gender, smoking, BMI, high BP, and taking prescription medication. Overall, the data provide additional support to the thesis that indoor air pollution is an important modifiable factor in the risk of hypertension. Future studies should be conducted over an extended period of time and include other important indoor air pollutants such as volatile organic compounds and formaldehyde.

## Figures and Tables

**Table 1:** Summary of participants by indoor PM_2.5_ concentration (based on all subjects *n* = 63).

Variables	Overall (*n* = 63)	Missing Cases *n* (%)	PM_2.5_ * (µg/m^3^)		
			<25	≥25	*p*^§^ Value
Age (years)	61 (9.00)	0 (0)	61 (9.30)	63 (9.50)	0.306
BMI (kg/m^2^)	28.71 ± 5.64	6 (9.52)	28.36 ± 6.29	29.28 ± 4.51	0.569
Born in Australia					
Yes	34 (54.00)	0 (0)	20 (66.70)	10 (33.30)	0.849
No	29 (46.00)		18 (64.30)	10 (35.70)	
Smokers in house					
Yes	4 (6.30)	0 (0)	3 (75.00)	1 (25.00)	1.000
No	59 (93.7)		35 (64.80)	19 (35.20)	
Drinker					
Yes	8 (12.70)	0 (0)	6 (85.70)	1 (14.30)	0.403
No	55 (87.30)		32 (62.70)	19 (37.30)	
Diabetes					
Yes	20 (31.70)	0 (0)	9 (50.00)	9 (50.00)	0.095
No	43 (68.30)		29 (72.50)	11 (27.50)	
Hypertensive					
Yes	20 (32.70)	2 (3.17)	13 (68.40)	6 (31.60)	0.790
No	41 (65.10)		24 (64.90)	13 (35.10)	
Prescription medicine					
Yes	31 (49.2)	0 (0)	19 (65.50)	10 (34.50)	
No	32 (50.8)		19 (65.50)	10 (34.50)	1.000
HR (bpm)	63.25 (12.63)	11 (17.46)	62.5 (12.38)	65 (13.50)	0.214
SBP (mmHg)	130.25 ± 17.69	11 (17.46)	128.00 ± 16.51	133.85 ± 19.32	0.250
DBP (mmHg)	75.12 ± 9.98	11 (17.46)	73.88 ± 10.20	77.10 ± 9.52	0.261
McAuley’s ISI	8.40 ± 2.65	15 (23.81)	8.27 ± 2.67	8.59 ± 2.69	0.694

Continuous variables (age, BMI, HR, SBP, DBP and McAuley’s ISI) are presented as mean ± standard deviation (SD) if symmetrical or as median (IQR) if skewed. Categorical variables (born in Australia, smoking, drinker, diabetes, hypertensive, prescription medicine) are presented as *n* (%). ^§^ *p*-value correspond to an independent samples *t*-test (symmetrical continuous data), or a Mann-Whitney U test (skewed continuous data), or either a chi-square test or Fishers’ exact test if applicable (categorical data). * Categorized based on WHO guidelines (2005). BMI: body mass index; HR: heart rate; SBP: systolic blood pressure; DBP: diastolic blood pressure; ISI: McAuley’s Insulin Sensitivity Index.

**Table 2:** Summary statistics for indoor PM concentration, temperature and relative humidity (*n* = 63).

Variable *	Mean	SD	Median	IQR	Min	Max
PM_1_ (µg/m^3^)	17.62	20.23	6.00	32.00	0.00	62.00
PM_2.5_ (µg/m^3^)	18.74	19.92	7.00	31.00	1.00	62.00
PM_4_ (µg/m^3^)	21.91	21.83	8.00	31.50	2.00	71.00
PM_10_ (µg/m^3^)	37.29	22.49	34.50	38.00	10.00	95.00
PM_Total_ (µg/m^3^)	66.16	30.50	63.00	32.25	23.00	168.00
Temperature (°C)	23.09	3.54	23.42	5.09	16.52	32.16
Relative humidity (%)	53.24	13.13	54.84	15.87	0.64	84.70

* Five (7.94%) households had missing values for all variables. IQR: interquartile range.

**Table 3:** Unit changes (95% CI) in outcome variables associated with a one interquartile range (IQR) increase in indoor PM concentration exposures—for all subjects (*n* = 63).

Outcome Variable	*n*	Indoor Air Quality (µg/m^3^)				
		PM_1_ (IQR = 32)	PM_2.5_ (IQR = 31)	PM_4_ (IQR = 31.5)	PM_10_ (IQR = 38)	Total PM (IQR = 32.25)
HR (bpm)						
CModel	52	2.93 (−0.89, 6.99)	3.04 (−0.72, 7.02)	3.80 * (0.34, 7.45)	4.47 * (0.37, 8.83)	3.00 * (0.49, 5.61)
AModel	45	4.56 * (0.45, 8.93)	4.38 * (0.31, 8.71)	3.13 (−1.31, 7.88)	3.39 (−2.44, 9.78)	0.91 (−3.46, 5.60)
SBP (mmHg)						
CModel	52	1.43 (−5.87, 8.73)	1.65 (−5.53, 8.82)	1.77 (−4.89, 8.44)	3.38 (−4.43, 11.18)	2.94 (−1.90, 7.78)
AModel	45	1.63 (−6.26, 9.53)	1.75 (−6.05, 9.55)	−0.31 (−8.74, 8.11)	3.86 (−7.11, 14.83)	5.89 (−2.25, 14.04)
DBP (mmHg)						
CModel	52	0.32 (−3.78, 4.43)	0.45 (−3.59, 4.49)	1.83 (−1.88, 5.55)	2.24 (−2.13, 6.62)	2.01 (−0.69, 4.71)
AModel	45	2.04 (−2.11, 6.18)	2.03 (−2.07, 6.13)	1.96 (−2.46, 6.38)	2.70 (−3.10, 8.50)	1.57 (−2.85, 5.98)

Crude model: adjusted for age and gender. Adjusted model: adjusted for age, gender, BMI, smokers in house, hypertensive, prescription medicine, and McAuley’s ISI. For the HR variable, natural logarithmic transformation was used. The estimated coefficients corresponding to the expected geometric mean of the HR were then back transformed using an exponential function * *p* < 0.05.

**Table 4:** Unit changes (95% CI) in outcome variables associated with one IQR increase in indoor PM concentration exposures—for non-hypertensive subjects (*n* = 41).

Outcome Variable	*n*	Indoor Air Quality (µg/m^3^)				
		PM_1_ (IQR = 30.50)	PM_2.5_ (IQR = 30)	PM_4_ (IQR = 31)	PM_10_ (IQR = 33)	Total PM (IQR = 32.25)
HR (beat/minute)						
CModel	33	5.43 * (0.05, 11.26)	5.67 * (0.35, 11.44)	6.03 * (1.09, 11.35)	6.05 * (1.11, 11.37)	4.96 ^ 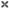 ^ (1.66, 8.43)
AModel	30	6.15 * (1.32, 11.34)	6.03 * (1.18, 11.24)	4.55 (−0.87, 10.43)	4.33 (−1.50, 11.81)	4.17 (−1.90, 10.84)
SBP (mmHg)						
CModel	33	4.55 (−3.64, 12.73)	4.80 (−3.31, 12.91)	3.90 (−3.78, 11.56)	7.16 ^♌^ (−0.19, 14.51)	6.97 ^ 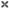 ^ (2.16, 11.79)
AModel	30	6.32 (−2.44, 15.08)	6.40 (−2.34, 15.15)	3.65 (−6.06, 13.37)	10.01 ^♌^ (−0.61, 20.63)	13.44 ^ 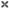 ^ (4.07, 22.81)
DBP (mmHg)						
CModel	33	2.92 (−1.79, 7.64)	3.15 (−1.58, 7.85)	4.13 ^♌^ (−0.13, 8.38)	4.67 * (0.50, 8.85)	3.69 * (0.84, 6.54)
AModel	30	3.58 (−1.58, 8.73)	3.61 (−1.55, 8.76)	3.64 (−1.93, 9.21)	5.32 ^♌^ (−1.01, 11.64)	4.64 (−1.48, 10.76)

Crude model: adjusted for age and gender. Adjusted model: adjusted for age, gender, BMI, smokers in house, hypertensive, prescription medicine, and McAuley’s ISI. For the HR variable, natural logarithmic transformation was used. The estimated coefficients corresponding to the expected geometric mean of the HR were then back transformed using an exponential function. ^
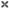
^ *p* < 0.01, * *p* < 0.05, ^♌^ *p* < 0.1.

## Supplementary Materials

The following are available online at http://www.mdpi.com/1660-4601/15/9/2026/s1, Figure S1: Percentage changes (95% CI) in outcome variables associated with one interquartile range (IQR) increase in PM concentration exposures ^¥^—for all subjects (*n* = 63), Figure S2: Percentage changes (95% CI) in outcome variables associated with one interquartile range (IQR) increase in PM concentration exposures ^¥^—for subjects who answered “no” to “high blood pressure” (*n* = 41).
