# Supplementary material for: The Association between Indoor Air Quality and Adult Blood Pressure Levels in a High-Income Setting
Source: Int J Environ Res Public Health. 2018 Sep 17;15(9):2026. doi: 10.3390/ijerph15092026 (PMC6164223; doi:10.3390/ijerph15092026)
Supplement: Supplementary file 1 [file ijerph-15-02026-s001.pdf]

Figure S1. Percentage changes (95% CI) in outcome variables associated with one interquartile range (IQR) increase in PM concentration exposures—for all subjects (n = 63).

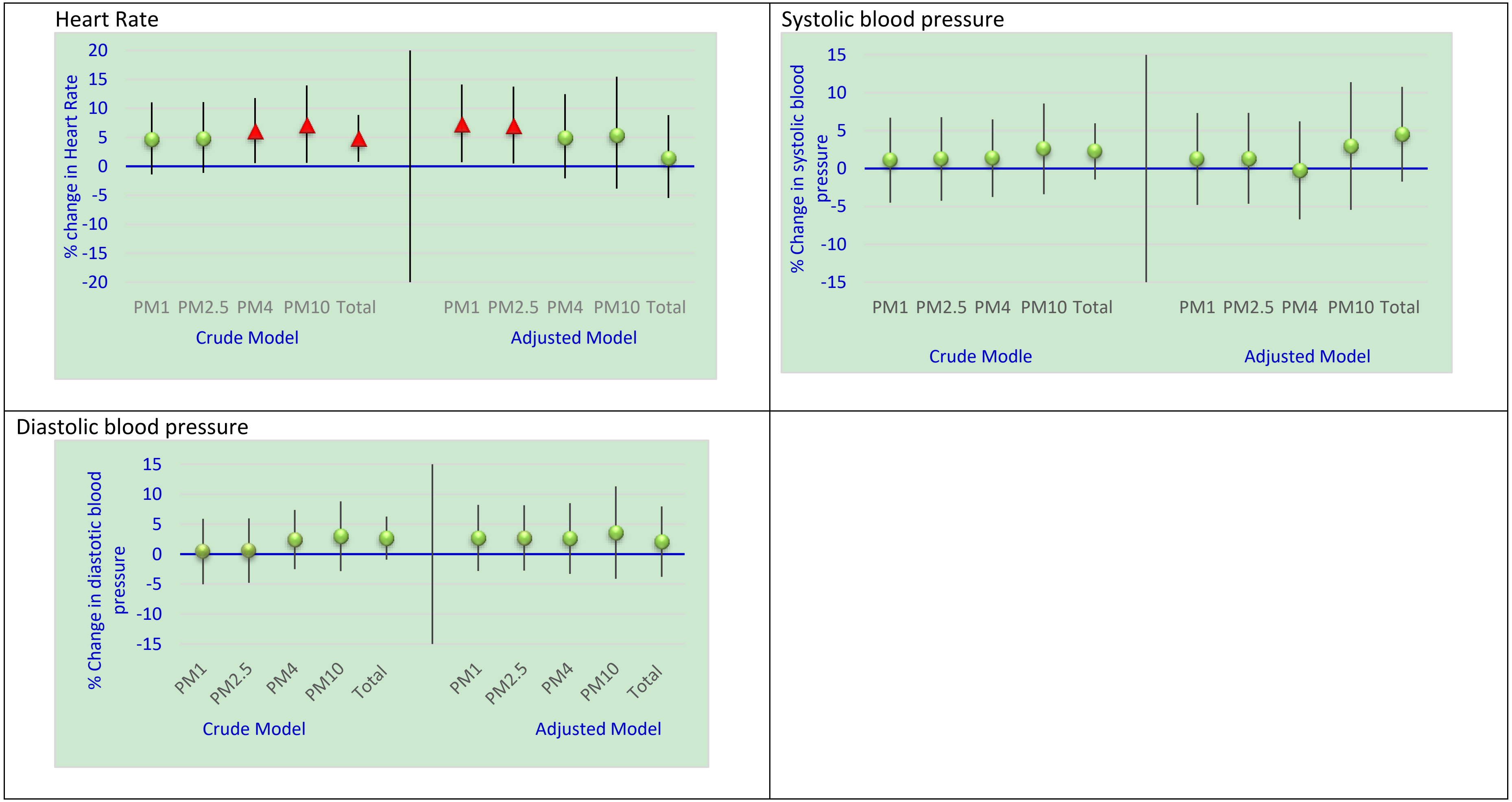

Crude model: adjusted for age and gender. Adjusted model: adjusted for age, gender, BMI, smokers in house, hypertensive, prescription medicine, and McAuley's ISI.

The natural logarithm transformed variable was used and the estimated coefficients corresponding to the expected geometric mean of the heart rate were back transformed using an exponential function.

: no significant change; : increase at least at 10% significance level ($p < 0.1$); : increase at least at 5% significance level ($p < 0.05$); : increase at least at 1% significance level ($p < 0.01$). PM: particulate matter.

Int. J. Environ. Res. Public Health **2018**, *15*, 2026; doi:10.3390/ijerph15092026

Figure S2. Percentage changes (95% CI) in outcome variables associated with one interquartile range (IQR) increase in PM concentration exposures—for subjects who answered "no" to "high blood pressure" ($n = 41$).

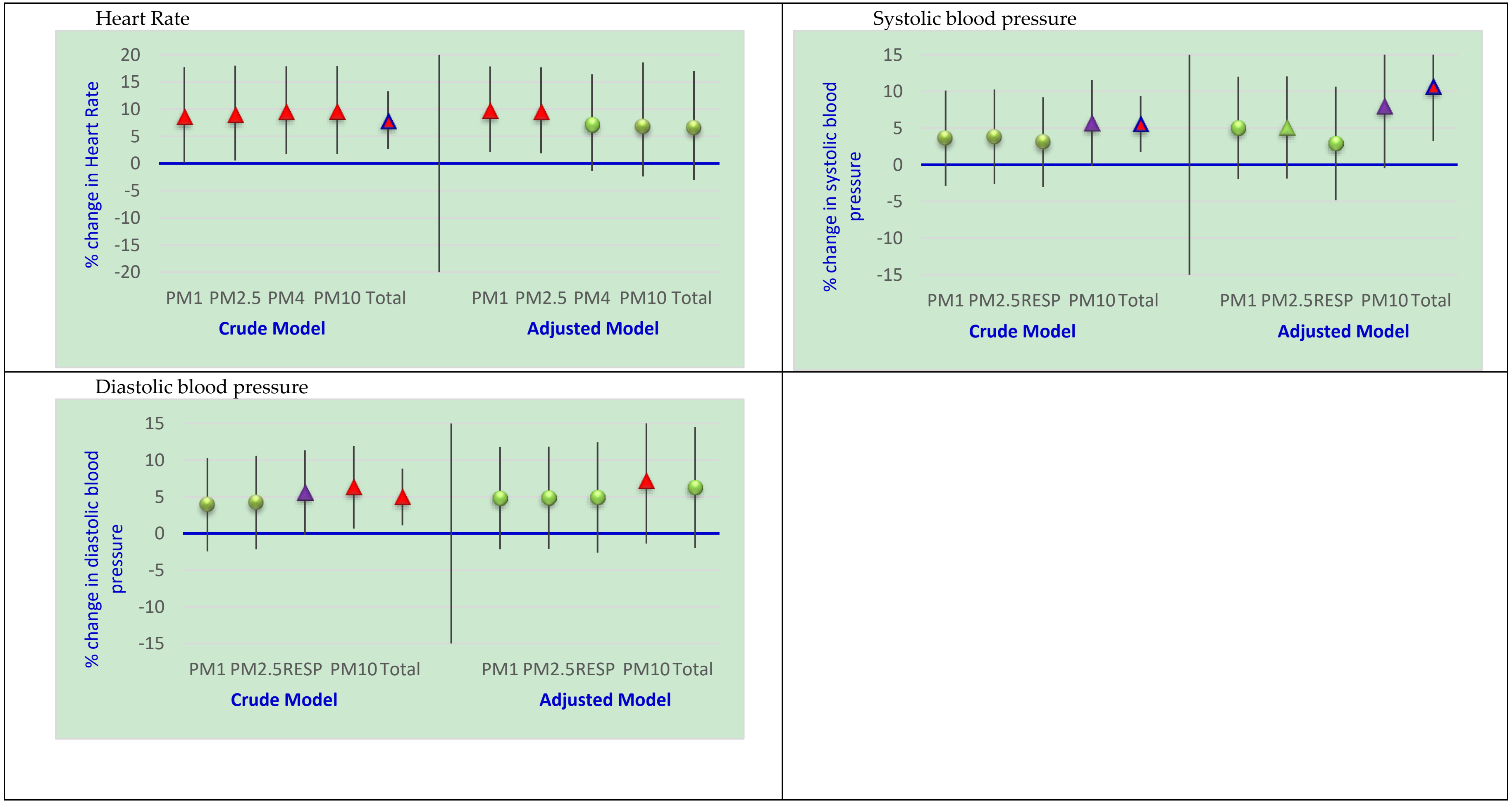

Crude model: adjusted for age and gender. Adjusted model: adjusted for age, gender, BMI, smokers in house, prescription medicine, and McAuley's ISI. The natural logarithm transformed variable was used and the estimated coefficients corresponding to the expected geometric mean of the heart rate were back transformed using an exponential function.

●: no significant change; ▲: increase at least at 10% significance level ($p < 0.1$); ▲: increase at least at 5% significance level ($p < 0.05$); ▲: increase at least at 1% significance level ($p < 0.01$)
